# Human Gut Microbiota in Heart Failure: Trying to Unmask an Emerging Organ

**DOI:** 10.3390/biomedicines11092574

**Published:** 2023-09-19

**Authors:** Ioannis Paraskevaidis, Andrew Xanthopoulos, Elias Tsougos, Filippos Triposkiadis

**Affiliations:** 1 6th Department of Cardiology, Hygeia Hospital, 15123 Athens, Greece; 2 Department of Cardiology, University Hospital of Larissa, 41110 Larissa, Greece; andrewvxanth@gmail.com (A.X.); ftriposkiadis@gmail.com (F.T.)

**Keywords:** heart failure, gut, microbiota, relationship

## Abstract

There is a bidirectional relationship between the heart and the gut. The gut microbiota, the community of gut micro-organisms themselves, is an excellent gut-homeostasis keeper since it controls the growth of potentially harmful bacteria and protects the microbiota environment. There is evidence suggesting that a diet rich in fatty acids can be metabolized and converted by gut microbiota and hepatic enzymes to trimethyl-amine N-oxide (TMAO), a product that is associated with atherogenesis, platelet dysfunction, thrombotic events, coronary artery disease, stroke, heart failure (HF), and, ultimately, death. HF, by inducing gut ischemia, congestion, and, consequently, gut barrier dysfunction, promotes the intestinal leaking of micro-organisms and their products, facilitating their entrance into circulation and thus stimulating a low-grade inflammation associated with an immune response. Drugs used for HF may alter the gut microbiota, and, conversely, gut microbiota may modify the pharmacokinetic properties of the drugs. The modification of lifestyle based mainly on exercise and a Mediterranean diet, along with the use of pre- or probiotics, may be beneficial for the gut microbiota environment. The potential role of gut microbiota in HF development and progression is the subject of this review.

## 1. Introduction

Heart failure (HF) is a severe and harmful syndrome and although many diagnostic and therapeutic efforts have been made, effective, holistic management has not yet been achieved. The reported data suggest an HF prevalence ranging from 1% to 2% in adults [1,2,3] and the HF incidence seems to be higher and increases with age, exceeding 10% for those >70 years old [3,4]. Importantly, the mortality rate is high [5,6] and is expected to increase further due to the increased population, aging, senescence, coexisting morbidities, and probably the lack of holistic prevention and management [7]. Thus, although HF is common, its morbidity and mortality rates remain high [8]. Of note, despite the fact that the major determinants of syndrome severity, namely, prolonged activation of neurohormonal systems, inflammation, and free radical production, have been recognized and the relevant treatments have been implemented, there are still several important issues to be resolved. Indeed, when referring to the HF process, diverse additional factors adversely affecting body homeostasis should be considered [9,10]. It has long been recognized [11,12] that HF by inducing gut ischemia and congestion may alter the gut microbiota (the community of gut micro-organisms themselves) and intestinal permeability, stimulating immune and inflammatory processes [13,14] and leading to a further deterioration of cardiac function [15,16]. Moreover, as the gut microbiota regulates the energetic function of several organs, including the heart, its derangement may be associated with multiorgan dysfunction [17,18,19].

## 2. Bidirectional Relationship between the Heart and the Gut

**Figure 1:**
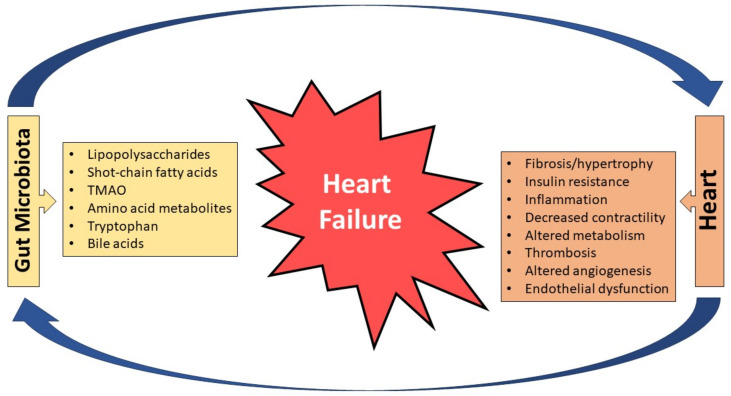
The reciprocal association between the gut microbiota and its products and cardiac dysfunction. Gut microbiota and cardiac dysfunction contribute to a negative spiral leading to heart failure development and progression; TMAO: trimethylamine N-oxide.

It is well known that neurohormonal activation in HF (activation of the sympathetic nervous system {SNS} and renin-angiotensin-aldosterone system {RAAS}) is initiated as a compensatory response to hemodynamic instability (decreased cardiac output and increased filling pressures) but eventually leads to multiorgan hypoperfusion and dysfunction (liver, kidneys, gut, etc.). In this regard, gut ischemia and edema promote gut barrier dysfunction accompanied by an intestinal leak of microbes and/or their products, facilitating their entrance into circulation (“leaky gut” hypothesis of HF) and causing low-grade inflammation together with a relevant immune response (Figure 1). A decrease in perfusion particularly affects the structure of villi in the intestinal mucosa, which is susceptible to ischemia due to the countercurrent circulation present in the villus (the artery and vein have a parallel trajectory), leading to a descending tissue gradient in oxygen pressure and/or tension from the base to the tip of the villus. Intramucosal acidosis has been reported in approximately 50% of patients with decompensated HF and thickening of the intestinal wall along with edema can be seen in HF patients. Notably, the amount of collagen in their mucosal walls is increased, commensurate with the HF severity [20].

Conversely, the derangement of microbiota and development of one with abnormal endocrine and homeostatic regulatory characteristics [21] facilitates insulin resistance, obesity, and metabolic syndrome [22] suggesting that the gut micro-organism composition is strongly associated with cardiovascular diseases [16,17,23,24]. The gut microbiota converts complex carbohydrates to short-chain fatty acids in order to enhance intestinal absorption [25]. The gut microbial metabolites may lead to weight loss by affecting appetite hormones, reducing fat accumulation with the suppression of triacylglycerols and up-regulation of adipose tissue browning, improving gut mucosal function and hormonal secretion, and alleviating gut inflammation. At the same time, dysbiosis may expand short-chain fatty acid-producing bacteria, which can be utilized as an additional energy source. Furthermore, increased Gram-negative bacteria can produce excess lipopolysaccharides, which in turn increase gut paracellular permeability, triggering endotoxemia and low-grade systemic inflammation. This rise in body fat, along with a potential increase in systemic inflammation may result in increased epicardial fat dysregulation, which has been associated with unfavorable cardiovascular outcomes [26]. Changes in gut microbiota and its products share various pathways with those of HF, activating directly or indirectly the immune, neurohumoral, and inflammatory processes [27,28,29] and, therefore, contributing to left ventricular (LV) remodeling and myocardial fibrosis [15,30,31]. Interestingly, epicardial adipose tissue may represent a key contributor to the development of HF with preserved left ventricular ejection fraction, and, therefore, is an appealing therapeutic target [26].

It has been suggested that the gut microbiota affects non-myocyte cardiac cells. In particular, a hypercaloric diet may decrease the expression of proteins located in the intestinal wall between enterocytes and allow for the passage of lipopolysaccharides, which in turn act on the type 4 toll receptors which are located in adipose tissue, resulting in the activation of the nuclear translocation of nuclear factor kappa B (NF-kB) and the subsequent release of pro-inflammatory substances contributing to the classic low-grade inflammation seen in “sick” (dysfunctional) adipose tissue [32]. Furthermore, gut microbiota metabolites, namely, short-chain fatty acids, play a key role in energy metabolism and immunomodulation by acting on free fatty acid receptor 2 (FFAR2) and free fatty acid receptor 3 (FFAR3), which are located in the gastrointestinal system, nervous system, and adipose tissue. Thus, gut dysbiosis in obese individuals may result in changes in short-chain fatty acid levels and, by extension, “sick” adipose tissue-related metabolic alterations [32]. Higher short-chain fatty acid production has been suggested to promote lipogenesis. Typical characteristics of the “sick” adipose tissue observed in obese patients include altered angiogenesis and endothelial dysfunction [32]. Lastly, in cardiac fibroblasts, trimethylamine N-oxide (TMAO), another metabolic end-product of the gut microbiota, enhances transforming growth factor β (TGFβ) receptor I expression and inhibits the expression of SMAD2, a downstream inhibitor of TGFβ signaling. By facilitating TGFβ signaling, TMAO promotes cardiac fibroblast differentiation into myofibroblasts, causing cytokine secretion and cardiac fibrosis [33]. Some of the HF microbiome (the collective genomes of the gut micro-organisms) patterns are similar to those observed in diverse clinical settings such as cardiometabolic disease [30], inflammatory bowel disease, and several other chronic diseases [13,31]. Thus, considering the emerging role of the human gut microbiota and its bidirectional association with the heart, the exploration and better understanding of this emerging organ is mandatory [34,35,36,37,38].

## 3. Understanding the Gut Microbiota

The human gut contains approximately 100 trillion micro-organisms, which correspond to 5000 different species and weigh roughly 2 kg [39]. The gut microbiota composition includes bacteria, viruses, fungi, and parasites, with the main species of bacteria being *Prevotella*, *Bacteroidetes*, *Ruminococcus*, and *Firmicutes*. In the human adult, *Firmicutes* are the most frequent, followed by *Bacteroidetes* and *Actinobacteria* [6], and the proportion between the bacterial species *Bacteroidetes* and *Firmicutes* seems to have a crucial role in health and disease. Bacteria from the gut microbiome participate in the harvesting of energy from food, regulate the beneficial and opportunistic bacterial composition, and produce neurotransmitters, such as serotonin, enzymes, and vitamins [40].

At the time of birth, the intestinal tract is rather sterile but becomes rapidly colonized by trillions of non-pathogenic organisms affected mainly by environmental factors. The composition of this new organ is not uniform and is personalized and, therefore, differs from individual to individual, depending on the host’s genetic variation, diet, lifestyle, xenobiotics, and medications [41,42,43,44]. In fact, in different individuals under the same dietary regimen, blood glucose levels vary depending on microbiota composition [45]. It seems that the microbiota is altered by dietary habits [41] but it is unknown whether dietary interventions can modify cardiovascular risk by affecting microbiota composition [31]. Nevertheless, the production of short-chain fatty acids from gut microbiota improves intestinal barrier function, modulates blood pressure, inhibits inflammation, and contributes to the regulation of the epigenome balance and immunity response as well. Acetate-producing bacteria seem to play a pivotal role in cardiac hypertrophy and fibrosis [46]. Interestingly, it seems that the gut composition contributes to the body mass index [47] and the development of autoimmune diseases in humans [48]. The bile acid pool, which is altered in patients with HF, closely interacts with the gut microbiota [49] and exhibits a negative chronotropic effect on the myocardium [50,51], which has been attributed to the inverse relation of bile acid to β-adrenoreceptor activity and affinity [52]. Indeed, it has been demonstrated that the bile acid receptor orphan nuclear receptor-FXR through nuclear factor-kB, [53,54] promotes cardiac hypertrophy [55,56], facilitates apoptosis through mitochondrial signaling [57], and modulates metabolism and inflammation [58].

The Na+/H+ exchange (NHE) is a membrane transport mechanism that belongs to members of the cation/proton antiporters superfamily. NHE utilizes energy reserved as an electrochemical Na+ concentration gradient across the plasma membrane by the basolateral Na+/K+-adenosine triphosphatase [59]. NHE is an important mechanism for the transepithelial transfer of Na+ and HCO_3_^−^ and supports other nutrient transporters by supplying the proton gradient for the proton-coupled absorption of amino acids, peptides, organic anions, short-chain fatty acids, and iron. NHE2, NHE3, and NHE8 have been involved in the control of the fundamental functions of epithelial cells, such as the regulation of intracellular pH, cell volume, and nutrient absorption, as well as in cell proliferation, cell migration, and apoptosis [59,60,61]. Interestingly, the inhibition of intestinal NHE3 activity has emerged as a promising treatment for hypertension [59].

Lifestyle habits affect the gut microbiota. Exercise reduces cytokine levels [62], sleep disorders affect the microbiota community, and stress alters intestinal permeability [63]. Working conditions, sexual habits, and physical interactions also affect the microbiota composition [64,65,66]. Since the gut microbiota is influenced by several factors, the gut composition is characterized both by stability and dynamic variation, which is an obstacle to its use as a biomarker [67,68,69,70]. Unfortunately, the main body of relevant evidence originates from animal models, rendering doubtful extrapolations to humans [31,71]. Whether the longitudinal control of microbiota variations can give us information regarding HF and its management remains to be elucidated. Despite these limitations, there is evidence suggesting that a diet rich in fatty acids (e.g., choline and L-Carnitine) can be metabolized and converted by gut microbiota and hepatic enzymes to TMAO, a product associated with atherogenesis, platelet dysfunction, thrombotic events, coronary artery disease, stroke, HF, and ultimately death [72,73,74,75,76,77,78]. In patients with chronic HF, the increase in the levels of TMAO is associated with ventricular dysfunction and decreased survival [79,80,81,82]. A study that included 1208 patients with chronic HF after myocardial infarction reported that the major adverse cardiovascular event (MACE) risk increased with an elevation in TMAO levels, and this positive correlation became more significant when TMAO levels were higher than the median [83]. In the same study, TMAO was also found to be an independent predictor of all-cause mortality after adjusting for traditional risk factors. The same seems to be true in acute HF, although for the time being, there are only reports originating from experimental models [84,85]. Finally, a meta-analysis of 12 studies including 13,425 participants demonstrated that, compared to low-level TMAO, increased TMAO was associated with MACEs and all-cause mortality in HF; consistent results were observed in all examined subgroups and the sensitivity analysis [86].

In patients with HF, several comorbidities are also present, including kidney dysfunction, iron deficiency and anemia, diabetes, electrolyte disorders, and obesity, which may affect the microbiota composition. An interesting study by Cui et al. reported a decrease in *Faecalibacterium prausnitzii* (one of the most abundant butyrate-producing species exerting anti-inflammatory actions) and an increase in *Ruminococcus gnavus* (a bacterium with pro-inflammatory properties) in HF patients [87]. Furthermore, a comprehensive analysis of microbiota by Kummen et al. reaffirmed the decreased microbial diversity in HF, identified changes in fifteen core taxa, and emphasized a depletion of the Lachnospiraceae family (of which several members are butyrate producers) which was inversely associated with increased levels of soluble CD25, a marker for T-cell and macrophage activation [35]. Concerning kidney dysfunction, a relationship between kidney function, cardiovascular disease, and gut microbiota has been suggested [88,89,90]. Iron deficiency and anemia, which are associated with high cardiovascular and all-cause mortality [91,92], are also controlled by gut microbiota to some extent [93,94,95,96,97]. The increased morbidity and mortality observed in diabetes, insulin resistance, and obesity are partially dependent on gut microbiota status. Of note, obesity, a sedentary lifestyle, genetic susceptibility [98] and, ultimately, gut microbiota dysbiosis (“imbalance” in the gut micro-organism community that is associated with disease) seem to contribute to the development of T2DM [99,100]. A study that compared 291 non-diabetic Danish individuals with 75 individuals with T2DM showed that the increased levels of branched-chain amino acids in diabetic individuals correlated with the gut microbiota, suggesting that microbiota configurations contribute to the development of insulin resistance and pointing out that targeting these microbial clusters may have the potential to diminish insulin resistance and reduce the incidence of common metabolic and cardiovascular disorders [101].

## 4. Gut Microbiota as a Diagnostic Marker

According to the National Institute of Health (NIH), a biomarker is defined as “a characteristic that is objectively measured and evaluated as an indicator of normal biologic processes, pathogenic processes, or pharmacologic responses to a therapeutic intervention” [102]. Alterations in gut flora have been linked to several human diseases, including gastrointestinal disorders [103], ischemic stroke [104], allergies [105,106], inflammation [107,108,109], cancer [110,111,112,113] and cardiovascular disease [114,115,116]. For example, gut microbiota derangement is linked to ST-elevation myocardial infarction [117] and can be used in the setting of a relevant prediction model [118]. Thus, it is reasonable to search for gut microbiota alterations per se or its products as diagnostic disease biomarkers [119,120,121].

As previously mentioned, there is a relationship between gut microbiota, neuro-hormonal activity, inflammation, and free oxygen production, the steadfast underpinnings of HF [35,36,38]. It is reasonable, therefore, based on this relationship, to test microbiota-based biomarkers in the diagnosis and management of HF.

TMAO is the most studied microbiota biomarker, showing a correlation with the HF functional class [79,80,81,122] and with the B-type natriuretic peptide, with mortality either in chronic [122] or acute HF [84]. Interestingly, the correlation between TMAO and mortality remains even after adjustment for the natriuretic peptide levels [79,123]. Additionally, TMAO can be used as an index of mortality/hospitalization risk in HF patients with a preserved ejection fraction presenting with restrictive physiology patterns [79,84,124,125,126]. Finally, it seems that TMAO can be used as an index of cardiac fibrosis and contractility, platelet reactivity, and endothelial function [13].

It has been suggested that short-chain fatty acids augment mitochondrial DNA protection and regulate ATP concentration, thus, controlling the energetic needs of several organs, including the heart [17,125,127,128,129]. They are inversely correlated with the outcomes in HF patients with reduced ejection fraction [35] and can be used as markers of cardiac fibrosis and hypertrophy [46,130,131], vascular tone [46,130,131], gut barrier function [132], and insulin sensitivity [133]. Given their efficiency and the observation that the enzymatic machinery for oxidation of short-chain fatty acids is up-regulated in the failing hearts of both animals and humans, targeting this unexplored source of energy for therapy for patients with HF could be a promising area of future clinical studies [134]. Nevertheless, their effects always depend on which receptor and in which tissue/cell type they activate each time. FFAR3 and FFAR2 are mainly short-chain fatty acid receptors [135]. Contrary to FFAR2, whose role in cardiovascular homeostasis is virtually unknown, FFAR3’s involvement in cardiovascular function regulation has become increasingly clear over the past decade. FFAR3 has been implicated in the mechanism of lipolysis, while also exerting vasodilating properties resulting in hypotension. On the other hand, FFAR3 promotes neuronal firing and norepinephrine synthesis and release in sympathetic neurons [136] and increases heart rate and cardiac inflammation [135]. Lipopolysaccharides consisting of a hydrophobic domain known as lipid A (or endotoxin), a non-repeating “core” oligosaccharide, and a distal polysaccharide (or O-antigen) are elevated in decompensated HF [137] and play a crucial role in gut barrier function, inflammation, cardiac contractility, insulin resistance, and endothelial function.

Phenylacetyl glutamine (PAGln) along with phenylacetylglycine (PAGly) are gut microbiota metabolites that act through G-protein coupled receptors and are involved in platelet function and thrombosis, leading, therefore, to cardiovascular disease [34,138]. Their presence in blood samples is related to increased reactive oxygen production and apoptosis, decreased cell viability and myocardial contraction, and high rates of thrombotic events [138,139,140]. The increased free radical production activates the enzyme calmodulin kinase II (CaMKII) and the ryanodine receptor 2 (RyR2), inducing a proarrhythmic status characterized by cardiomyocyte apoptosis and electrical remodeling [141,142]. Indeed, a recently conducted study demonstrated that plasma PAGln levels are significantly elevated in atrial fibrillation, suggesting that PAGln may be a promising therapeutic target in this clinical setting [140].

Based on the above, it is tempting to suggest the use of gut microbiota or their metabolites either in feces or in blood samples as biomarkers of cardiovascular involvement. However, there are several limitations, mainly because the normal microbiota has not been adequately defined [38]. Additionally, both gut microbiota composition and its products as well as HF are influenced by age [143]. Moreover, there is a database limitation for studying the human gut microbiome [144] and the coupling of taxonomy and function in the microbiome is not well defined. It is hoped that these discrepancies could be resolved [145] by using artificial intelligence, *16S rRNA* gene sequencing, or even whole metagenome shotgun sequencing [146,147].

## 5. Gut Microbiota and Medications

**Figure 2:**
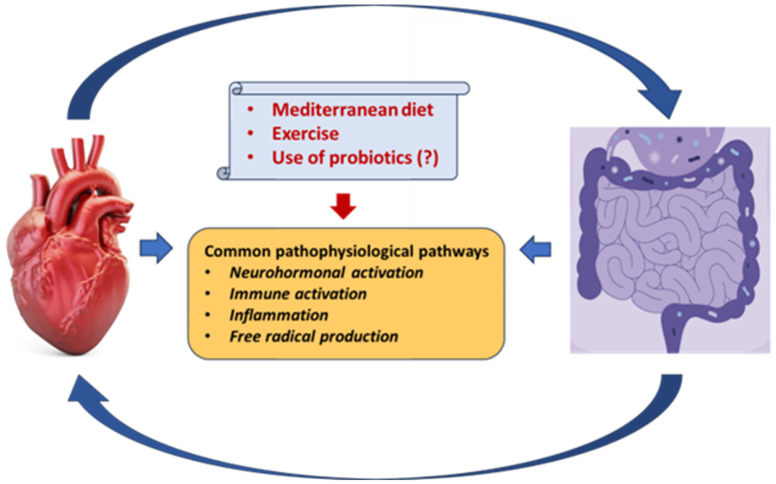
Common pathophysiological pathways between the gut microbiota and the heart in heart failure. A Mediterranean diet, exercise, and possibly the use of probiotics may attenuate these dangerous interactions.

There is a bidirectional relationship between the gut microbiota and drugs since microbiota can be altered by drug action and, conversely, the microbiota can modify the pharmacokinetic properties of drugs. Resistance to aspirin [148], along with other platelet aggregation inhibitors, due to microbiota action has also been documented [149]. The use of proton pump inhibitors has been associated with an increase in typically oral bacteria in the gut [150,151]. Metformin, an antidiabetic drug, has been associated with changes in the gut microbiome composition both in vivo and in mice [152,153]. An in vitro analysis of more than 1000 marketed drugs revealed that non-antibiotic drugs can also inhibit the growth of gut bacterial strains [154]. Further, most of the drugs used in HF, including β-blockers, angiotensin receptor blockers, angiotensin-converting enzyme inhibitors, calcium channel blockers, statins, and the more recently introduced SGLT2 inhibitors [155], can alter the gut microbiota composition [149,156,157], which in turn may modify drug action and ultimately affect HF management. An interesting multi-drug meta-analysis of three independent Dutch cohorts (*N* = 2396 individuals) reported that the administration of proton pump inhibitors, laxatives, and antibiotics had the largest effect on gut microbiome composition [158]. Although there is a well-documented bidirectional relationship between gut microbiota and medications, the exact mechanisms underlying this interaction have not been delineated. However, there is some evidence to suggest that lifestyle modifications including exercise and a Mediterranean diet, along with the use of pre- or probiotics, might beneficially alter the gut microbiota environment [122,159,160,161] (Figure 2). Current evidence, however, is insufficient, and new paths of research are required to explore new approaches for treatment optimization. Machine learning prediction tools have been developed for investigating the possibility of drug degradation by gut microbes [162]. For example, a machine learning model, trained on over 18,600 drug-bacteria interactions, has been recently proposed to predict (Area Under the Receiver Operating Curve of 0.857) whether drugs would impair the growth of 40 gut bacterial strains [163]. Sequencing the gut microbial genome could also be an option, but it is still under investigation [117,125,164,165]. In the meantime, antibiotics, bile acid sequestrants, non-lethal microbial inhibitors, fecal microbiota transplantation, etc. [37,166] might be used along with necessary lifestyle changes.

## 6. Gut Microbiota, Aging, Diet, Exercise Training, and Supplements

Aging, an inevitable evolution in all species, is characterized by the progressive functional deterioration of multiple organs that leads to dysfunctional tissues, with the cardiovascular system being no exception. Several studies, which have been performed in order to find an approach to extending life span, suggest that life duration depends on the type of diet, exercise, working environment, and pharmacological intervention [167,168]. It is well known that adherence to a Mediterranean diet provides a positive trajectory toward healthy successful aging, with major potential benefits for mental and cognitive health [169]. A study that included 153 subjects following the Mediterranean diet reported an increase in the level of fecal short-chain fatty acids, indicating a close relationship between this type of diet and a beneficial gut microbiota profile [170]. The effect of diet on microbiota and health was also demonstrated in another study that included 178 elderly subjects (>65 years); it was reported that the fecal microbiota composition was significantly associated with measures of frailty and comorbidity, as well as markers of inflammation [171]. In the same work, the individual microbiota of people in long-stay care was less diverse compared with that of community dwellers, and the loss of community-associated microbiota was related to increased frailty. Finally, an experimental study in mice showed that a high-fat, high-sugar diet promoted metabolic disease by depleting Th17-inducing microbes, and recovery of commensal Th17 cells restored protection [172]. Thus, a diet with moderate protein consumption, low glycemic index, and abundance of foods rich in fibers and polyphenols, may promote normal gut symbiosis and, hence, healthy aging.

Along with a healthy diet, several studies have suggested the beneficial effect of exercise on the intestinal flora [173]. Indeed, it has been shown that the gut microbiota affects the exercise capacity both of trained and not trained individuals, being a regulatory factor of the physiological function of skeletal muscles [174]. Further, regular exercise training beneficially affects the human lipid profile, metabolic status, and immune activity, reducing the risk for cardiovascular diseases [173,175,176]. Concerning HF, there are diverging data regarding the effect of diet on cardiac function [177,178]. Although there is a large number of studies that recommend the use of a healthy diet, exercise training, and, in some cases, the use of supplements, the evidence is not robust enough to strongly recommend this approach. However, it is a fact that, whereas the consumption of non-refined fiber-rich foods, vegetables, fruits, etc. promotes short-chain fatty acid production, which is considered cardioprotective, meat consumption leads to TMAO production, which is considered harmful for various systems, including the cardiovascular system [84,159]. Importantly, a relation between gut microbiota and mitochondria has been documented [179], indicating that the gut environment regulates cell death by toxin secretion, targeting the mitochondria and host innate immune system and leading to chronic inflammation that, in turn, promotes the dysfunction of various systems, including the cardiovascular [17]. In this respect, by maintaining the gut microbiota “keeper” on track, the control of mitochondrial function and minimization of harmful effects might be achieved. To answer important questions on these issues the PROMOTe (PROtein and Muscle in Older Twins, NCT04309292) study was designed [180]. This is a double-blinded, randomized, placebo-controlled, dietary intervention study in which volunteers are enrolled in twin pairs from the TwinsUK cohort. Each pair is randomized to either receive protein supplementation plus placebo or protein supplementation plus a gut microbiome modulator and the intervention period will last 12 weeks. Clinical and biochemical measures will be collected at 0 and 12 weeks, with two monthly contacts where the gut microbiota composition will be examined, together with a battery of physical assessments. The primary outcome will include the muscle function estimated utilizing the chair-rise time.

A recent meta-analysis of 15 randomized controlled trials examining the differences in the gut microbiome composition between patients on antibiotic therapy with and without additional probiotic supplementation revealed no significant differences between the probiotic-supplemented and control groups [181]. Therefore, the authors concluded that probiotics have only a minor, not permanent effect on the composition of the gut microbiome during antibiotic therapy and are not appropriate for preventing dysbiosis due to antibiotics (Table 1) [181].

## 7. Future Directions

Gut dysbiosis (altered intestinal microbiota) is associated with several human diseases [197]. As a result, several relevant biomarkers have been proposed for early disease detection. However, due to the heterogeneity of the gut environment and the lack of a definition of a healthy gut microbiota, the current relevant biomarkers are imprecise, and, therefore, of doubtful significance for disease classification [198,199,200,201,202,203]. Encouraging, however, are the results from studies employing machine learning and artificial intelligence for the differentiation between normal and abnormal gut microbiota as well as the prediction of treatment response in diverse diseases [204,205,206]. A recently published study, which systematically investigated the cross-cohort efficiency of gut microbiota-based machine-learning classifiers for 20 diseases, announced high predictive accuracies in intra-cohort validation but low accuracies in cross-cohort validation, with the exception of intestinal diseases [207]. Other studies demonstrated that the assessment of the gut microbiota status using machine learning/artificial intelligence may be useful in staging and the response to the treatment of cancer [208,209]. Unfortunately, this is currently not the case in HF. It is anticipated, however, that computational methods assessing the gut microbiota status will prove effective in HF early diagnosis, disease monitoring, and the evaluation of treatment response, contributing to the better management of this lethal syndrome in the not-so-distant future.

## *8.* Conclusions

The gut microbiota is an emerging organ that exhibits a bidirectional association with the heart and deserves our attention. Diet, exercise, and the use of medications may modify the gut microbiota composition and its interactions with several crucial pathophysiological mechanisms of HF. Whether the evaluation of gut microbiota may prove useful for HF early diagnosis, monitoring, and management is currently a subject of intensive research.

## Figures and Tables

**Table 1:** Main probiotic and antibiotic trials for examining gut–heart interactions.

Study	Country	Study Design *	Population		Antibiotics (and Additional) Treatment			Probiotic Supplementation	
			Number of Randomized Patients (Female %)	Age (Years—Mean ± SD) in the Intervention (and Control) Groups	Indication	Type	Duration (Days)	Type	Duration (Days)
Cárdenas et al. (2020) [182]	Ecuador	Single-blinded RCT	38 (60.5)	37.9 ± 7.2 (39.5 ± 10.7)	Helicobacter pylori infection	Amoxicillin, tinidazole, and omeprazole	14	*Saccharomyces boulardii*	14
Chen et al. (2018) [183]	China	Open-label RCT	70 (78.5)	43.89 ± 12.50 (43.20 ± 12.45)	Helicobacter pylori infection	Pantoprazole, amoxycillin, furazolidone, and colloidal bismuthpectin	14	*Clostridium butyricum*	14
De Wolfe et al. (2018) [184]	USA	Double-blinded, placebo-controlled RCT	31 (N.D.)	N.D. (N.D.)	Clostridioides difficile infection	Vancomycin, metronidazole, or fidaxomicin	28	*Lactobacillus acidophilus*, *Lactobacillus* *paracasei*, *Bifidobacterium lactis* Bi-07, and *Bifidobacterium lactis* Bi-04	28
Kabbani et al. (2017) [185]	USA	Open-label RCT	24 (59)	N.D. (N.D.)	Healthy volunteers—no indication	Amoxycillin-clavulanate	7	*Saccharomyces boulardii*	14
Kakiuchi et al. (2020) [186]	Japan	Open-label RCT	65 (44.6)	15.31 ± 0.32 (15.08 ± 0.28)	Helicobacter pylori infection	Vonoprazan, amoxycillin, and clarithromycin	7	*Enterococcus faecium*	7
MacPherson et al. (2018) [187]	Canada	Double-blinded, placebo-controlled RCT	70 (N.D.)	N.D. (N.D.)	Healthy volunteers—no indication	Amoxycillin trihydrate, potassium clavulanate	7	*Lactobacillus rhamnosus* and *Lactobacillus helveticus*	14
Oh et al. (2016) [188]	Korea	RCT	20 (30)	51.7 ± 0.79 (49.3 ± 3.56)	Helicobacter pylori infection	Clarithromycin, Amoxycillin, Lansoprazole	14	*Streptococcus faecium* and *Bacillus subtilis*	14
Tang et al. (2021) [189]	China	Placebo-controlled multicenter RCT	151 (34.4)	43.29 ± 11.30 (45.32 ± 10.98)	Helicobacter pylori infection	Esomeprazole, amoxycillin, furazolidone, and bismuth potassium citrate	14	*Enterococcus faecium* and *Bacillus subtilis*	28
Zhong et al. (2021) [190]	China	Open-label parallel RCT	42 (52.4)	All neonates (All neonates)	15 neonates with neonatal pneumonia, 5 neonates with urinary tract infection, and 35 neonates with non-specific infection	Piperacillin-tazobactam	7	*Bifidobacterium longum*, *Lactobacillus acidophilus* and *Enterococcus faecalis*	7
Engelbrektson et al. (2009) [191]	USA	Placebo-controlled RCT	40 (77.5)	36.5 ± N.D. (39.5 ± N.D.)	Healthy volunteers—no indication	Amoxicillin and clavulanic acid	7	*Bifidobacterium lactis* BI-04, *Bifidobacterium lactis* Bi-07, *Lactobacillus acidophilus*, *Lactobacillus paracasei* and *Bifidobacterium bifidum*	21
Forssten et al. (2014) [192]	Finland	Double-blinded, parallel RCT	80 (50)	33.7 ± 9.4 (30.9 ± 10.3)	Healthy volunteers—no indication	Amoxicillin and clavulanate	7	*Lactobacillus acidophilus*, *Bifidobacterium animalis* ssp. Lactis	14
Madden et al. (2005) [193]	UK	Pilot-scale, double-blinded RCT	13 (53.8)	60 ± N.D. (49 ± N.D.)	Helicobacter pylori infection	Amoxicillin, metronidazole, and lansoprazole	8	*Lactobacillus acidophilus* and 2 strains of *Bifidobacterium bifidum*	14
Plummer et al. (2005) [194]	UK	Double-blinded RCT	155 (N.D.)	N.D. N.D.	Helicobacter pylori infection	Amoxicillin, clarithromycin, and lansoprazole	7	*Lactobacillus acidophilus* and 2 strains of *Bifidobacterium* spp.	21
Wang et al. (2017) [195]	China	Double-blinded RCT	20 (45)	37.1 ± 12.3 (42.8 ± 13.8)	Helicobacter pylori infection	Esomeprazole, amoxicillin, clarithromycin, and tinidazole	14	*Saccharomyces boulardii*	14
Amarri et al. (2008) [196]	Italy	Open-label, national, parallel RCT	58 (50)	40 ± 18.9 months (42.1 ± 18.9 months)	Bacterial upper respiratory tract infections	Amoxicillin	5–10	Antibiotic-resistant *Bacillus clausii*	12–17

Abbreviations: RCT, randomized controlled rrial; USA, the United States of America; UK, the United Kingdom; N.D., No Data. * If not otherwise mentioned, the studies were single centers. Modified with permission from ref. [181].

## Data Availability

Not applicable.
